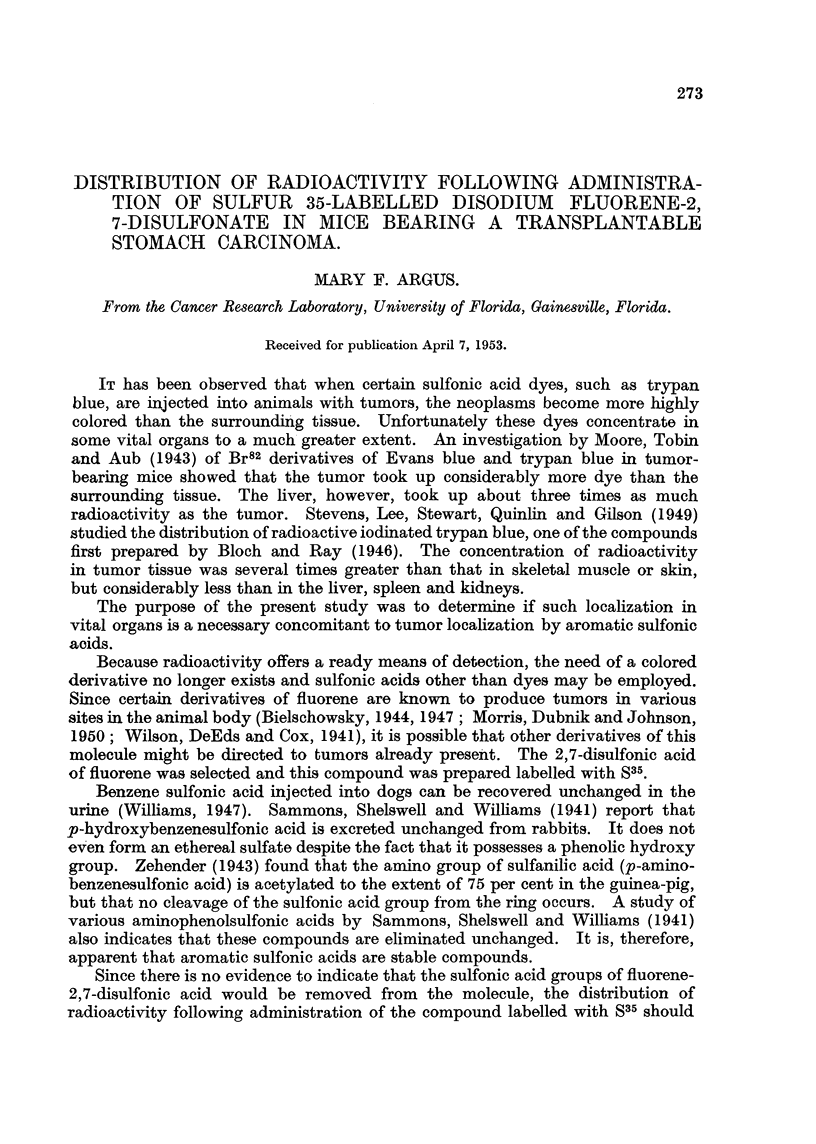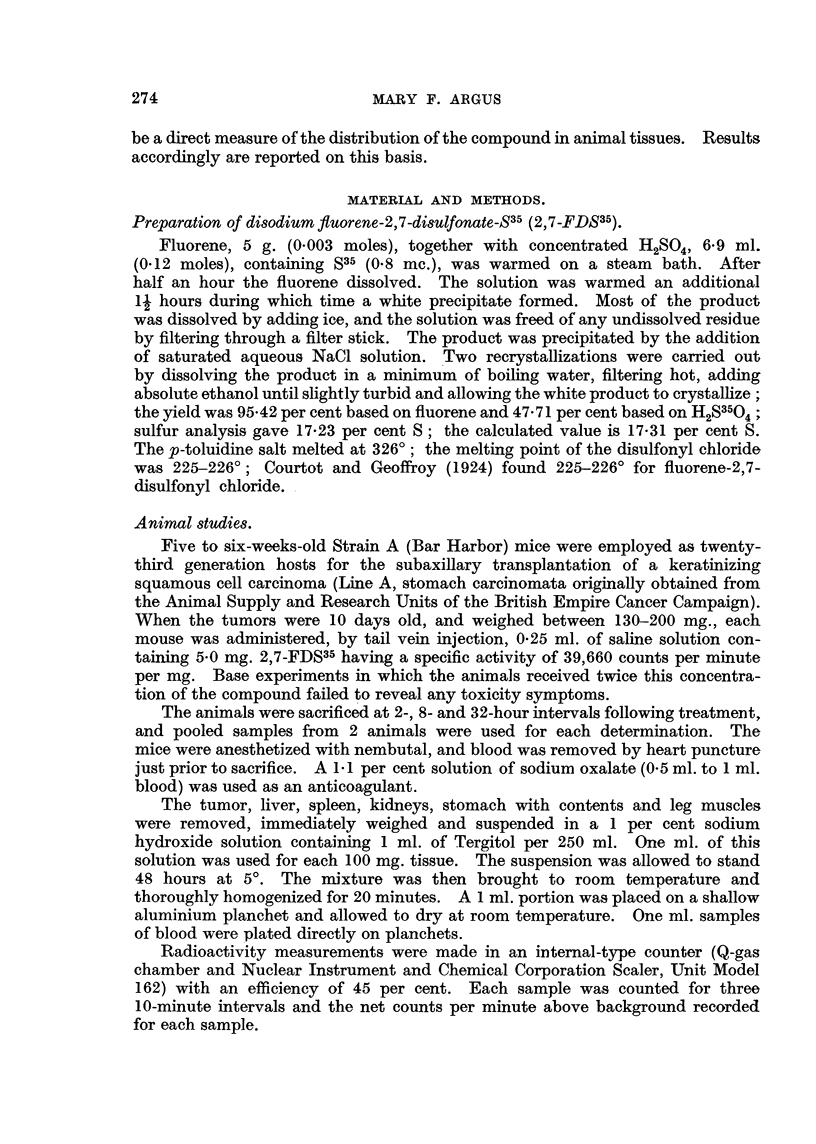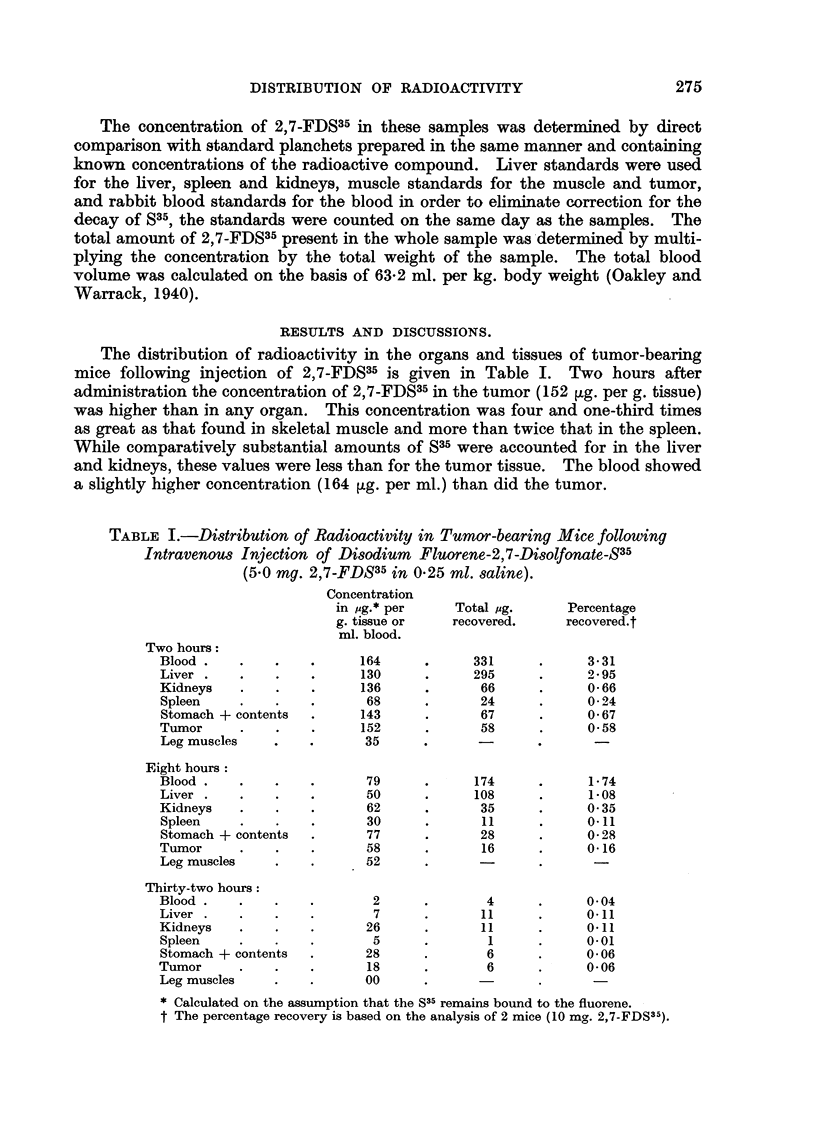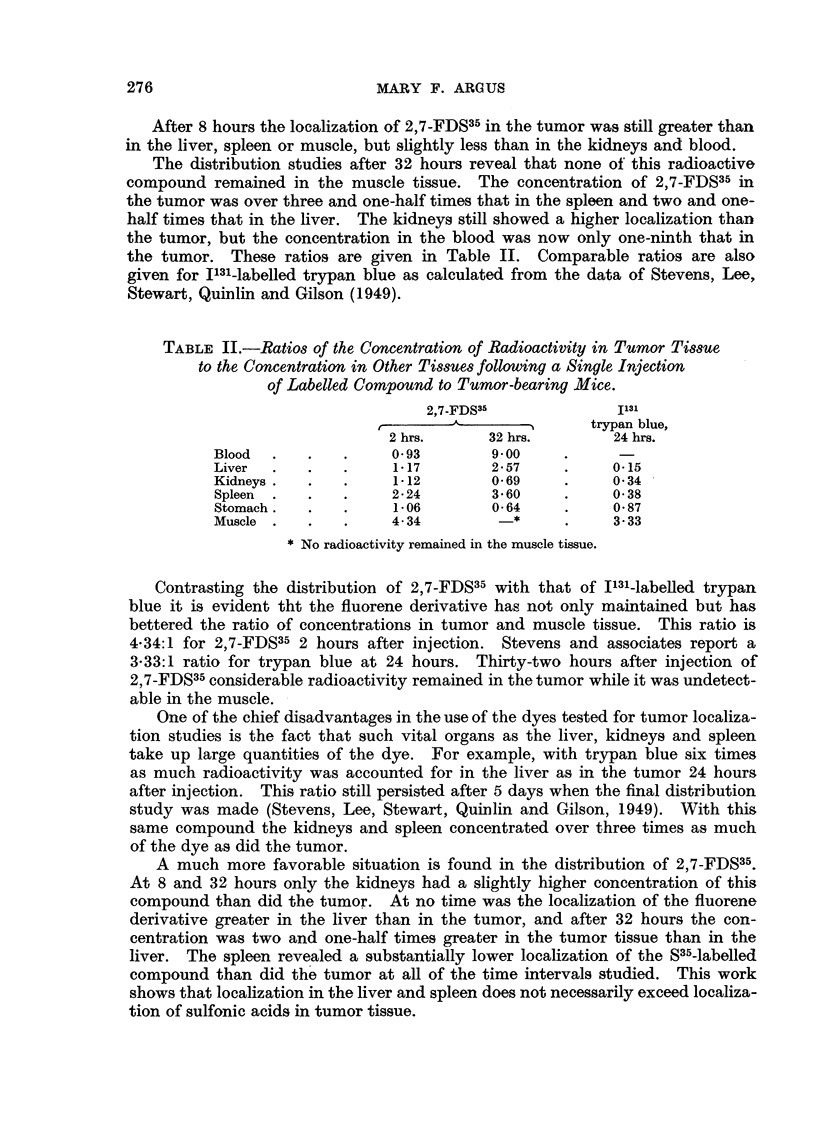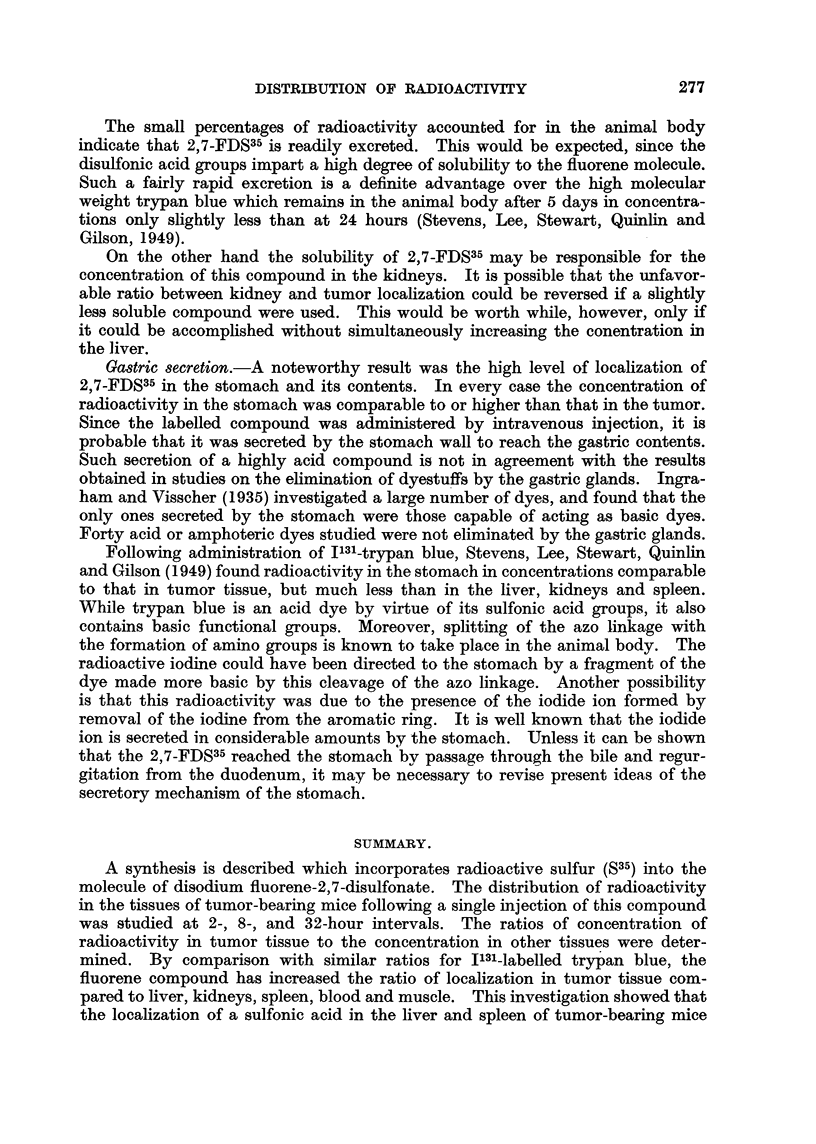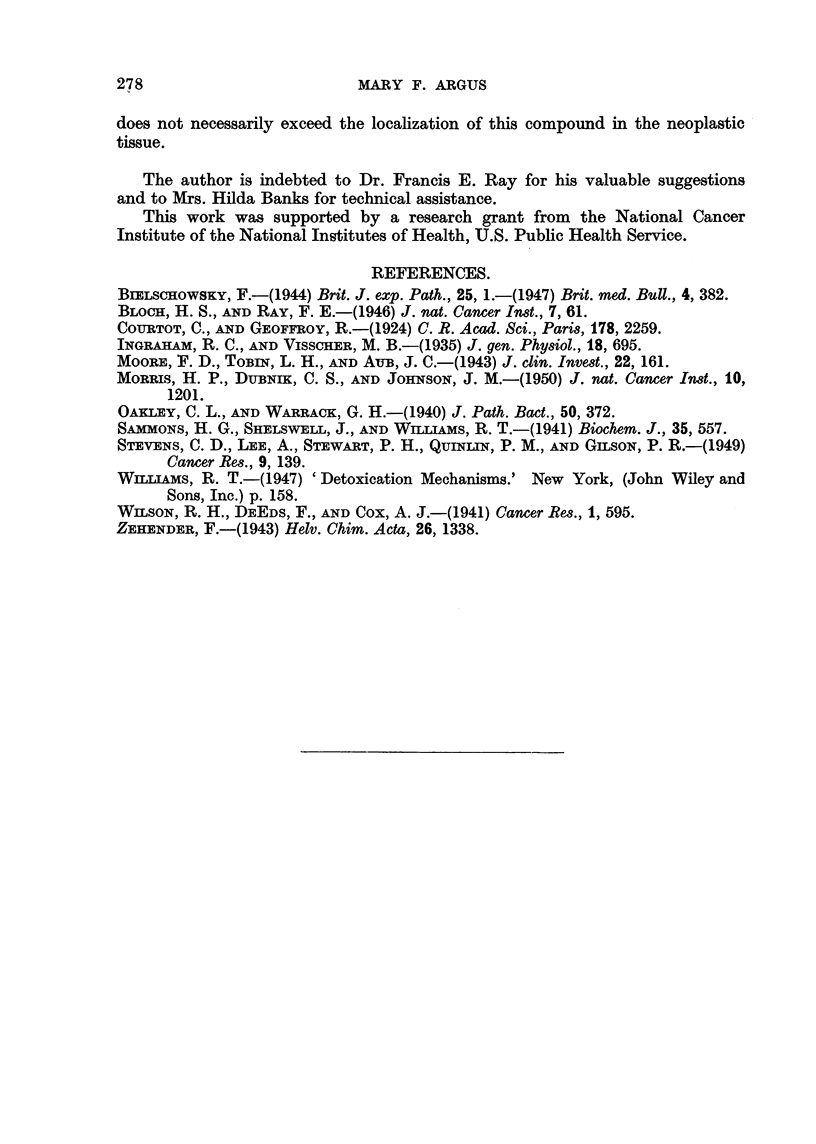# Distribution of Radioactivity following Administration of Sulfur 35-Labelled Disodium Fluorene-2, 7-Disulfonate in Mice Bearing a Transplantable Stomach Carcinoma

**DOI:** 10.1038/bjc.1953.26

**Published:** 1953-06

**Authors:** Mary F. Argus


### DISTRIBUTION OF RADIOACTIVITY FOLLOWING ADMINISTRATION OF SULFUR 35-LABELLED DISODIUM FLUORENE-2, 7-DISULFONATE IN MICE BEARING A TRANSPLANTABLE STOMACH CARCINOMA.

MARY F. ARGUS.

*From the Cancer Research Laboratory, University of Florida, Gainesville, Florida.*

Received for publication April 7, 1953.

It has been observed that when certain sulfonic acid dyes, such as trypan blue, are injected into animals with tumors, the neoplasms become more highly colored than the surrounding tissue. Unfortunately these dyes concentrate in some vital organs to a much greater extent. An investigation by Moore, Tobin and Aub (1943) of $Br^{82}$ derivatives of Evans blue and trypan blue in tumor-bearing mice showed that the tumor took up considerably more dye than the surrounding tissue. The liver, however, took up about three times as much radioactivity as the tumor. Stevens, Lee, Stewart, Quinlin and Gilson (1949) studied the distribution of radioactive iodinated trypan blue, one of the compounds first prepared by Bloch and Ray (1946). The concentration of radioactivity in tumor tissue was several times greater than that in skeletal muscle or skin, but considerably less than in the liver, spleen and kidneys.

The purpose of the present study was to determine if such localization in vital organs is a necessary concomitant to tumor localization by aromatic sulfonic acids.

Because radioactivity offers a ready means of detection, the need of a colored derivative no longer exists and sulfonic acids other than dyes may be employed. Since certain derivatives of fluorene are known to produce tumors in various sites in the animal body (Bielschowsky, 1944, 1947 ; Morris, Dubnik and Johnson, 1950 ; Wilson, DeEds and Cox, 1941), it is possible that other derivatives of this molecule might be directed to tumors already present. The 2,7-disulfonic acid of fluorene was selected and this compound was prepared labelled with $S^{35}$.

Benzene sulfonic acid injected into dogs can be recovered unchanged in the urine (Williams, 1947). Sammons, Shelswell and Williams (1941) report that *p*-hydroxybenzenesulfonic acid is excreted unchanged from rabbits. It does not even form an ethereal sulfate despite the fact that it possesses a phenolic hydroxy group. Zehender (1943) found that the amino group of sulfanilic acid (*p*-aminobenzenesulfonic acid) is acetylated to the extent of 75 per cent in the guinea-pig, but that no cleavage of the sulfonic acid group from the ring occurs. A study of various aminophenolsulfonic acids by Sammons, Shelswell and Williams (1941) also indicates that these compounds are eliminated unchanged. It is, therefore, apparent that aromatic sulfonic acids are stable compounds.

Since there is no evidence to indicate that the sulfonic acid groups of fluorene-2,7-disulfonic acid would be removed from the molecule, the distribution of radioactivity following administration of the compound labelled with $S^{35}$ should be a direct measure of the distribution of the compound in animal tissues. Results accordingly are reported on this basis.

### MATERIAL AND METHODS.

*Preparation of disodium fluorene-2,7-disulfonate-$S^{35}$* (2,7-*FDS*$^{35}$).

Fluorene, 5 g. (0·003 moles), together with concentrated $H_2SO_4$, 6·9 ml. (0·12 moles), containing $S^{35}$ (0·8 mc.), was warmed on a steam bath. After half an hour the fluorene dissolved. The solution was warmed an additional 1½ hours during which time a white precipitate formed. Most of the product was dissolved by adding ice, and the solution was freed of any undissolved residue by filtering through a filter stick. The product was precipitated by the addition of saturated aqueous NaCl solution. Two recrystallizations were carried out by dissolving the product in a minimum of boiling water, filtering hot, adding absolute ethanol until slightly turbid and allowing the white product to crystallize ; the yield was 95·42 per cent based on fluorene and 47·71 per cent based on $H_2S^{35}O_4$ ; sulfur analysis gave 17·23 per cent S ; the calculated value is 17·31 per cent S. The *p*-toluidine salt melted at 326° ; the melting point of the disulfonyl chloride was 225–226° ; Courtot and Geoffroy (1924) found 225–226° for fluorene-2,7-disulfonyl chloride.

*Animal studies.*

Five to six-weeks-old Strain A (Bar Harbor) mice were employed as twenty-third generation hosts for the subaxillary transplantation of a keratinizing squamous cell carcinoma (Line A, stomach carcinomata originally obtained from the Animal Supply and Research Units of the British Empire Cancer Campaign). When the tumors were 10 days old, and weighed between 130–200 mg., each mouse was administered, by tail vein injection, 0·25 ml. of saline solution containing 5·0 mg. 2,7-FDS$^{35}$ having a specific activity of 39,660 counts per minute per mg. Base experiments in which the animals received twice this concentration of the compound failed to reveal any toxicity symptoms.

The animals were sacrificed at 2-, 8- and 32-hour intervals following treatment, and pooled samples from 2 animals were used for each determination. The mice were anesthetized with nembutal, and blood was removed by heart puncture just prior to sacrifice. A 1·1 per cent solution of sodium oxalate (0·5 ml. to 1 ml. blood) was used as an anticoagulant.

The tumor, liver, spleen, kidneys, stomach with contents and leg muscles were removed, immediately weighed and suspended in a 1 per cent sodium hydroxide solution containing 1 ml. of Tergitol per 250 ml. One ml. of this solution was used for each 100 mg. tissue. The suspension was allowed to stand 48 hours at 5°. The mixture was then brought to room temperature and thoroughly homogenized for 20 minutes. A 1 ml. portion was placed on a shallow aluminium planchet and allowed to dry at room temperature. One ml. samples of blood were plated directly on planchets.

Radioactivity measurements were made in an internal-type counter (Q-gas chamber and Nuclear Instrument and Chemical Corporation Scaler, Unit Model 162) with an efficiency of 45 per cent. Each sample was counted for three 10-minute intervals and the net counts per minute above background recorded for each sample.

The concentration of 2,7-$FDS^{35}$ in these samples was determined by direct comparison with standard planchets prepared in the same manner and containing known concentrations of the radioactive compound. Liver standards were used for the liver, spleen and kidneys, muscle standards for the muscle and tumor, and rabbit blood standards for the blood in order to eliminate correction for the decay of $S^{35}$, the standards were counted on the same day as the samples. The total amount of 2,7-$FDS^{35}$ present in the whole sample was determined by multiplying the concentration by the total weight of the sample. The total blood volume was calculated on the basis of 63·2 ml. per kg. body weight (Oakley and Warrack, 1940).

### RESULTS AND DISCUSSIONS.

The distribution of radioactivity in the organs and tissues of tumor-bearing mice following injection of 2,7-$FDS^{35}$ is given in Table I. Two hours after administration the concentration of 2,7-$FDS^{35}$ in the tumor (152 µg. per g. tissue) was higher than in any organ. This concentration was four and one-third times as great as that found in skeletal muscle and more than twice that in the spleen. While comparatively substantial amounts of $S^{35}$ were accounted for in the liver and kidneys, these values were less than for the tumor tissue. The blood showed a slightly higher concentration (164 µg. per ml.) than did the tumor.

TABLE I.—*Distribution of Radioactivity in Tumor-bearing Mice following Intravenous Injection of Disodium Fluorene-2,7-Disolfonate-$S^{35}$ (5·0 mg. 2,7-$FDS^{35}$ in 0·25 ml. saline).*

| | Concentration in µg.* per g. tissue or ml. blood. | Total µg. recovered. | Percentage recovered.† |
|---|---|---|---|
| Two hours : | | | |
| Blood | 164 | 331 | 3·31 |
| Liver | 130 | 295 | 2·95 |
| Kidneys | 136 | 66 | 0·66 |
| Spleen | 68 | 24 | 0·24 |
| Stomach + contents | 143 | 67 | 0·67 |
| Tumor | 152 | 58 | 0·58 |
| Leg muscles | 35 | — | — |
| Eight hours : | | | |
| Blood | 79 | 174 | 1·74 |
| Liver | 50 | 108 | 1·08 |
| Kidneys | 62 | 35 | 0·35 |
| Spleen | 30 | 11 | 0·11 |
| Stomach + contents | 77 | 28 | 0·28 |
| Tumor | 58 | 16 | 0·16 |
| Leg muscles | 52 | — | — |
| Thirty-two hours : | | | |
| Blood | 2 | 4 | 0·04 |
| Liver | 7 | 11 | 0·11 |
| Kidneys | 26 | 11 | 0·11 |
| Spleen | 5 | 1 | 0·01 |
| Stomach + contents | 28 | 6 | 0·06 |
| Tumor | 18 | 6 | 0·06 |
| Leg muscles | 00 | — | — |

* Calculated on the assumption that the $S^{35}$ remains bound to the fluorene.
† The percentage recovery is based on the analysis of 2 mice (10 mg. 2,7-$FDS^{35}$).

After 8 hours the localization of 2,7-$FDS^{35}$ in the tumor was still greater than in the liver, spleen or muscle, but slightly less than in the kidneys and blood.

The distribution studies after 32 hours reveal that none of this radioactive compound remained in the muscle tissue. The concentration of 2,7-$FDS^{35}$ in the tumor was over three and one-half times that in the spleen and two and one-half times that in the liver. The kidneys still showed a higher localization than the tumor, but the concentration in the blood was now only one-ninth that in the tumor. These ratios are given in Table II. Comparable ratios are also given for $I^{131}$-labelled trypan blue as calculated from the data of Stevens, Lee, Stewart, Quinlin and Gilson (1949).

TABLE II.—*Ratios of the Concentration of Radioactivity in Tumor Tissue to the Concentration in Other Tissues following a Single Injection of Labelled Compound to Tumor-bearing Mice.*

| | 2,7-$FDS^{35}$ | | $I^{131}$ trypan blue, |
|---|---|---|---|
| | 2 hrs. | 32 hrs. | 24 hrs. |
| Blood | 0·93 | 9·00 | — |
| Liver | 1·17 | 2·57 | 0·15 |
| Kidneys | 1·12 | 0·69 | 0·34 |
| Spleen | 2·24 | 3·60 | 0·38 |
| Stomach | 1·06 | 0·64 | 0·87 |
| Muscle | 4·34 | —* | 3·33 |

* No radioactivity remained in the muscle tissue.

Contrasting the distribution of 2,7-$FDS^{35}$ with that of $I^{131}$-labelled trypan blue it is evident tht the fluorene derivative has not only maintained but has bettered the ratio of concentrations in tumor and muscle tissue. This ratio is 4·34:1 for 2,7-$FDS^{35}$ 2 hours after injection. Stevens and associates report a 3·33:1 ratio for trypan blue at 24 hours. Thirty-two hours after injection of 2,7-$FDS^{35}$ considerable radioactivity remained in the tumor while it was undetectable in the muscle.

One of the chief disadvantages in the use of the dyes tested for tumor localization studies is the fact that such vital organs as the liver, kidneys and spleen take up large quantities of the dye. For example, with trypan blue six times as much radioactivity was accounted for in the liver as in the tumor 24 hours after injection. This ratio still persisted after 5 days when the final distribution study was made (Stevens, Lee, Stewart, Quinlin and Gilson, 1949). With this same compound the kidneys and spleen concentrated over three times as much of the dye as did the tumor.

A much more favorable situation is found in the distribution of 2,7-$FDS^{35}$. At 8 and 32 hours only the kidneys had a slightly higher concentration of this compound than did the tumor. At no time was the localization of the fluorene derivative greater in the liver than in the tumor, and after 32 hours the concentration was two and one-half times greater in the tumor tissue than in the liver. The spleen revealed a substantially lower localization of the $S^{35}$-labelled compound than did the tumor at all of the time intervals studied. This work shows that localization in the liver and spleen does not necessarily exceed localization of sulfonic acids in tumor tissue.

The small percentages of radioactivity accounted for in the animal body indicate that 2,7-FDS$^{35}$ is readily excreted. This would be expected, since the disulfonic acid groups impart a high degree of solubility to the fluorene molecule. Such a fairly rapid excretion is a definite advantage over the high molecular weight trypan blue which remains in the animal body after 5 days in concentrations only slightly less than at 24 hours (Stevens, Lee, Stewart, Quinlin and Gilson, 1949).

On the other hand the solubility of 2,7-FDS$^{35}$ may be responsible for the concentration of this compound in the kidneys. It is possible that the unfavorable ratio between kidney and tumor localization could be reversed if a slightly less soluble compound were used. This would be worth while, however, only if it could be accomplished without simultaneously increasing the conentration in the liver.

*Gastric secretion.*—A noteworthy result was the high level of localization of 2,7-FDS$^{35}$ in the stomach and its contents. In every case the concentration of radioactivity in the stomach was comparable to or higher than that in the tumor. Since the labelled compound was administered by intravenous injection, it is probable that it was secreted by the stomach wall to reach the gastric contents. Such secretion of a highly acid compound is not in agreement with the results obtained in studies on the elimination of dyestuffs by the gastric glands. Ingraham and Visscher (1935) investigated a large number of dyes, and found that the only ones secreted by the stomach were those capable of acting as basic dyes. Forty acid or amphoteric dyes studied were not eliminated by the gastric glands.

Following administration of I$^{131}$-trypan blue, Stevens, Lee, Stewart, Quinlin and Gilson (1949) found radioactivity in the stomach in concentrations comparable to that in tumor tissue, but much less than in the liver, kidneys and spleen. While trypan blue is an acid dye by virtue of its sulfonic acid groups, it also contains basic functional groups. Moreover, splitting of the azo linkage with the formation of amino groups is known to take place in the animal body. The radioactive iodine could have been directed to the stomach by a fragment of the dye made more basic by this cleavage of the azo linkage. Another possibility is that this radioactivity was due to the presence of the iodide ion formed by removal of the iodine from the aromatic ring. It is well known that the iodide ion is secreted in considerable amounts by the stomach. Unless it can be shown that the 2,7-FDS$^{35}$ reached the stomach by passage through the bile and regurgitation from the duodenum, it may be necessary to revise present ideas of the secretory mechanism of the stomach.

### SUMMARY.

A synthesis is described which incorporates radioactive sulfur (S$^{35}$) into the molecule of disodium fluorene-2,7-disulfonate. The distribution of radioactivity in the tissues of tumor-bearing mice following a single injection of this compound was studied at 2-, 8-, and 32-hour intervals. The ratios of concentration of radioactivity in tumor tissue to the concentration in other tissues were determined. By comparison with similar ratios for I$^{131}$-labelled trypan blue, the fluorene compound has increased the ratio of localization in tumor tissue compared to liver, kidneys, spleen, blood and muscle. This investigation showed that the localization of a sulfonic acid in the liver and spleen of tumor-bearing mice does not necessarily exceed the localization of this compound in the neoplastic tissue.

The author is indebted to Dr. Francis E. Ray for his valuable suggestions and to Mrs. Hilda Banks for technical assistance.

This work was supported by a research grant from the National Cancer Institute of the National Institutes of Health, U.S. Public Health Service.

### REFERENCES.

BIELSCHOWSKY, F.—(1944) *Brit. J. exp. Path.*, **25**, 1.—(1947) *Brit. med. Bull.*, **4**, 382.
BLOCH, H. S., AND RAY, F. E.—(1946) *J. nat. Cancer Inst.*, **7**, 61.
COURTOT, C., AND GEOFFROY, R.—(1924) *C. R. Acad. Sci., Paris*, **178**, 2259.
INGRAHAM, R. C., AND VISSCHER, M. B.—(1935) *J. gen. Physiol.*, **18**, 695.
MOORE, F. D., TOBIN, L. H., AND AUB, J. C.—(1943) *J. clin. Invest.*, **22**, 161.
MORRIS, H. P., DUBNIK, C. S., AND JOHNSON, J. M.—(1950) *J. nat. Cancer Inst.*, **10**, 1201.
OAKLEY, C. L., AND WARRACK, G. H.—(1940) *J. Path. Bact.*, **50**, 372.
SAMMONS, H. G., SHELSWELL, J., AND WILLIAMS, R. T.—(1941) *Biochem. J.*, **35**, 557.
STEVENS, C. D., LEE, A., STEWART, P. H., QUINLIN, P. M., AND GILSON, P. R.—(1949) *Cancer Res.*, **9**, 139.
WILLIAMS, R. T.—(1947) 'Detoxication Mechanisms.' New York, (John Wiley and Sons, Inc.) p. 158.
WILSON, R. H., DEEDS, F., AND COX, A. J.—(1941) *Cancer Res.*, **1**, 595.
ZEHENDER, F.—(1943) *Helv. Chim. Acta*, **26**, 1338.